# Central Ohio Dental Association

**Published:** 1866-01

**Authors:** A. W. Maxwell


### Proceedings of Societies.

### CENTRAL OHIO DENTAL ASSOCIATION.

The first semi-annual meeting of the Central Ohio Dental Association was held at Wooster, on the 14th and 15th of November. The following members were present: Drs. Jas. Armstrong, Bucyrus; M. DeCamp, Mansfield; A. W. Maxwell, Galion; D W. Smith, Shelby; H. J. Cressinger, Ashland; J. M. Rhodes, Wooster; Wm. Mitchell, Mansfield; C. M. Kelsey, Mt. Vernon; and Wm. E. Dunn, Delaware. The following being duly proposed and elected were received as new members: Drs. J. B. Beauman, Columbus; D. B. Cressinger, Upper Sandusky; J. D. Moody, Dalton; H. C. Fowler, Columbus; J. W. R. Myers, Wooster, R. McDowell, Wooster; J. Boyer, Orrville; B. J. Jones, Wooster; B. F. Barclay, Dalton; H. M. Edson, Mt. Vernon; E. Chidester, Massillon; Jno. R. Suingly, Bucyrus; M. A. Spencer, Doylestown, and A. R. Lord, Shelby. The name of J. T. Wisner, Wooster, was duly proposed, but on ballot, he was rejected as being unworthy to become a member. Dr. Pierce, of Philadelphia, agent for Rubencame & Stockton, was elected an honorary member. The first session convened in Arcadome Hall at 1½ o'clock P. M. President, Dr. J. Armstrong in the chair, minutes of the last meeting read and approved. The committee appointed for the purpose at previous meeting, presented a set of by-laws and rules of business and order which were received and adopted.

An essay was read by Dr. H. J. Cressinger, subject, "The Province and duties of the Dentist," for which he received a vote of thanks, and a copy requested for publication in the "Dental Register."

Dr. Maxwell was gratified with the essay, and hoped there would be a free expression of opinion on subjects introduced in it. He urged the importance of regarding dentistry as a "fine art," and hoped more attention would be paid to obtaining artistic effects in inserting artificial teeth. By careful study of the case in hand, much of that mechanical stiffness and regularity, so common, could be avoided, and much of the natural contour and expression restored to the face; giving, in brief, some hints for accomplishing these results, by building on and increasing the thickness of the rubber work—particularly above the cuspids and molars to any desired fulness. As we must all render an account for the manner in which we have improved our knowledge and opportunities, it becomes us to be faithful and conscientious in the discharge of our responsibilities as dentists, objected to the term "job work," used in the essay, preferred mechanical or artificial dentistry.

Dr. DeCamp—did not wish to criticize the essay—was much pleased with it, and hoped it might have the effect to draw us together, and create a feeling of love for the profession, and good will toward our professional brethren: hoped every member would feel free to express his views on any subject suggested by the essay, and especially urged the young men to take hold of the work and overcome their timidity.

Dr. Boyer—was particularly pleased with the part of the essay, urging and encouraging friendly feelings among dentists. The mission of the dentist is to benefit humanity, and his responsibilities are great and his opportunities for doing good are not surpassed by those of any other class of professional men; he should therefore be a good man, honest and faithful in the discharge of his duties.

Dr. Kelsey—was just recovering from severe indisposition, had not been able to attend to business for several weeks, but could not stay away from this meeting; did not wish, or feel able, to talk much; hoped there would be a free discussion on any subject brought up ; would like to hear from Dr. McDowell, as the oldest member of the profession present, and as a representative of the vicinity in which our meeting is held.

Dr. McDowell—was quite unwell and did not wish to speak on the essay, but was much gratified to see the good feelings manifested here ; hoped and believed this association would accomplish much in creating a better state of feeling in the profession than has prevailed in our midst. Dr. Rhodes called attention to the subject of making fillings in cavities flooded with saliva, wished to hear the experience of members in regard to such operations.

Dr. Boyer—had no experience in making such fillings, has an inferior molar in his own mouth, which was filled by Prof. C. A. Harris, twelve years ago; was entirely flooded when the operation was done; is good and doing well yet.

Dr. McDowell—cleansed and formed the cavity as usual, and used a block or pellet of foil as large as could be introduced into the cavity, which he placed in position and packed firmly before it got wet, if possible, and then packed the best he could till full; sometimes succeeded quite well.

Dr. DeCamp's method was similar to Dr. McDowell's, but when the mouth filled with saliva, would allow the patient to empty it, and then proceed with the operation, always using non-adhesive gold in the wedging principle.

Dr. Kelsey—did not suppose it was designed to convey the idea to the younger members; that it was a good plan to make fillings under water ; thought we should use every precaution within our reach, to avoid and prevent the overflow of saliva; had never had good or satisfactory results in making submerged fillings—they would generally "crumble out beautifully."

Dr. Dunn—used the wedge and napkins to prevent the saliva from flowing into the cavity. In reply to question, did not find the wedge caused inflamation of the periosteum, used tinct. of arnica to prevent inflammation.

Dr. Beauman—used bibulous paper to close the ducts, napkins and the wedge, and when necessary the tongue compressor, and by these means succeeded very satisfactorily in keeping the mouth dry.

Dr. Maxwell—believed filling might be made of non-adhesive gold under water, which would be sufficiently compact to stay in and preserve the tooth for years, but did not think it practicable to make fillings of adhesive gold when wet, though, by way of experiment, had, ten years ago, filled a lower molar and built up a portion of the crown with sponge, nearly the whole operation done under water, the filling had preserved the tooth and is still pretty good.

Dr. DeCamp, in reply to question, preferred low numbers of foil.

Dr. Edson—thought more depended on having the instruments used for packing the gold in good condition, than in the number of the foil; they should be sharp and smooth; uses a plate of copper with oil and emery as a bone to dress the points of pluggers, to take off the wire edge made in filling. Adjourned to meet in the office of Rhodes and Myers at 6½ P. M.

EVENING SESSION.

Miscellaneous discussions continued.

Dr. Edson asked for the experience of members in different methods of annealing gold.

Dr. Rhodes—heated over stove and found the results more favorable than by any other method.

Dr. McDowell—used a thin plate of Platina to lay the gold on and heated over a lamp or on a stove.

Dr. DeCamp also used a Platina plate to heat the gold on —folded and cut the gold previous to heating, being careful to keep the pieces separate, or they will adhere to each other and be troublesome to handle.

After a considerable time spent in the election of members and the transaction of other business, the subject of deciduous teeth was taken up.

Dr. DeCamp considered it a subject of too great importance to be passed over in silence; thought it had received too little attention from parents and physicians, and even dentists. The deciduous teeth were generally regarded as of but little importance, as others would take their places when they were shed, or if painful from decay or any cause, they were extracted. Thought they should be retained in the mouth if possible, until the development of the permanent teeth required their removal; believed this end could be accomplished in most cases by proper treatment and filling; did not know how teeth, which were decayed to the gums and inflamed or ulcerated, or with the roots penetrating the gums excoriating the lip, could be saved or retained, and usually extracted them.

Dr Boyer—has practiced filling deciduous teeth with os-artificial with medium success. When pulp was exposed, sometimes filled over a cap and sometimes destroyed pulp and filled; but his success in such cases was not so good as he could desire; regarded it as being very important to save these teeth as long as possible, as cases occur where they are not shed till long after the usual time, and sometimes not at all. Mentioned a case he had recently seen, where both the deciduous and permanent teeth were in position in the arch, making a complete double row of teeth in the anterior portion of the mouth. Believed that diseases of the teeth were hereditary.

Dr. Dunn extracted childrens' teeth with great reluctance, thought much injury and sometimes permanent deformity, was occasioned by the too early extraction of these teeth.

Dr.Beauman—said, as a general thing, cases of diseases of childrens' teeth were presented to the dentist too late to admit of successful treatment for saving them—owing to ignorance of parents and physicians. Had recently had a case in his practice, where a physician had, for three months, treated a child for sore lip, caused by the roots of the incisors penetrating through the gums, without, in all that time, knowing the cause of the disease. He had effected a speedy cure by simply extracting the roots. Dentists should study the subject more, and improve every opportunity and use every means, within their reach, to enlighten parents and community in regard to the importance of giving care to childrens' teeth.

Dr. Armstrong—mentioned a case which had come under his observation, in which a child, at the age of a year and a half, had met with an accident, by which a number of teeth were knocked out, as the permanent teeth were never developed, he supposed their germs were so injured at that time as to stop their growth.

Adjourned to eight o'clock next morning.

SECOND DAY.

Met at 8 o'clock A. M. in the office of Drs. Rhodes and Myers. The attention of the association was first given to a clinic operation in filling, performed by Dr. Beauman, assisted by Dr. H. C. Fowler, in the use of the mallet, having for a subject our worthy President. The operation, which was a difficult one, was very skillfully performed. The Dr. used sponge gold, which he prefers for making adhesive fillings; he also accomplished the operation with explanations of his mode, and answered all inquiries of members, affording much valuable instruction to all who witnessed it. The remainder of the session was principally occupied in discussing the subject of dental fees, which resulted in the adoption, by the association, of a fee bill. On motion a special meeting was appointed to be held in Delaware, Delaware Co., on the 26th and 27th of Dec.* Adjourned at 12½ o'clock. All who were present seemed to feel that the meeting had been a very pleasant and profitable one.

A. W. Maxwell,
*Secretary.*

* By a vote of the society the Secretary was ordered to furnish a report of the proceedings of the meetings for publication in the "Dental Register."

